# Royal Hants County Hospital, Winchester

**Published:** 1909-12-04

**Authors:** J. Charles Warner

**Affiliations:** Chairman, and on behalf of the Committee of Management


### Editor's Letter-Box.

### ROYAL HANTS COUNTY HOSPITAL, WINCHESTER.

*To the Editor of* The Hospital.

Sir,—In your issue of October 16 there appeared an article by Sir Henry Burdett, which contained somewhat severe criticisms upon the management of this hospital.

The Managing Committee accepted those criticisms in the spirit in which we considered they were made, and although we did not, nor do we now, from an intimate knowledge of the circumstances admit they were justified, we thought it only courteous that they should receive some notice from us, and a reply was sent in a letter signed by myself as Chairman of the Committee, and which appeared in your paper of November 5.

In addition, however, to publishing that reply, you have added a footnote in which not only are the criticisms of Sir Henry Burdett supported, but remarks are added, the effect of which is to turn those criticisms (which of themselves suggested indifference on the part of the Committee) into what can only be considered as charges of neglect of duty and mismanagement.

This is intensified by the statement that representations bearing out these reflections have been made to you either by or on behalf of "influential Governors" who "agree with that report and consider a change in the present policy of the Committee essential to the best interests of this hospital."

It is open to suggestion that those Governors might much more effectually, and certainly more gracefully, have made these representations either to the Committee or at a Court of Governors, of which four are held each year.

The Committee therefore feel that, not only for themselves, as being persons who without in the least claiming any perfection, are at any rate willing to give both money and time in the service of the hospital, but also on behalf of those by whom they are elected they have a right to know on what authority these charges are made.

If they are founded upon the report of Sir Henry Burdett alone we say that the whole circumstances could not be sufficiently understood and appreciated in a flying visit of inspection and inquiry.

If they are made upon the representations of the Governors to whom you refer, then we submit that it is their plain duty as Governors of this Institution to come forward and substantiate their statements, in which case they have ample opportunity of laying them before a Court of Governors.

If no further and definite statement is forthcoming from them, then their representations must be treated in the only way in which it is possible to deal with charges of an anonymous nature.

Yours faithfully,
J. Charles Warner,
Chairman, and on behalf of the Committee
November 24, 1909. of Management.

[Mr. Warner misses the point of our comments on the letter which we published on the 6th ultimo. If he reads them again he will see that our remarks were directed against the present policy, only, of the Committee. The position is in a nutshell. The report of Sir Henry Burdett and our comments, together with the hospital itself, are open to the inspection of the governors, and, we presume, of all who are interested in hospital affairs. Mr. Warner has never denied the general accuracy of the comments and suggestions we have published with the sole object of bringing this hospital up to the standard of the best-administered modern hospitals. The Committee have it in their power to make this hospital up to date and efficient in every department. We imagine it must be their wish to do so; in which case the Committee and ourselves are in entire agreement. We must repeat, however, that the old policy of the Committee is not broad enough or wide enough, nor does it represent adequately the best spirit of hospital administration. This is the view which we stated was shared by influential governors who, believing, as we do, that the present Committee are well intentioned, hesitate to take action until the Committee have had the fullest opportunity of bringing the Royal Hants County Hospital up to date and making it thoroughly efficient in every department.—Ed. The Hospital.]

### "OFF THE LINE."

*To the Editor of* The Hospital.

